# Pathogenesis of Bone Alterations in Gaucher Disease: The Role of Immune System

**DOI:** 10.1155/2015/192761

**Published:** 2015-05-03

**Authors:** Juan Marcos Mucci, Paula Rozenfeld

**Affiliations:** IIFP, Departamento de Ciencias Biológicas, Facultad de Ciencias Exactas, Universidad Nacional de La Plata y CONICET, 47 y 115, 1900 La Plata, Argentina

## Abstract

Gaucher, the most prevalent lysosomal disorder, is an autosomal recessive inherited disorder due to a deficiency of glucocerebrosidase. Glucocerebrosidase deficiency leads to the accumulation of glucosylceramide primarily in cells of mononuclear-macrophage lineage. Clinical alterations are visceral, hematological, and skeletal. Bone disorder in Gaucher disease produces defects on bone metabolism and structure and patients suffer from bone pain and crisis. Skeletal problems include osteopenia, osteoporosis, osteolytic lesions, and osteonecrosis. On the other hand a chronic stimulation of the immune system is a well-accepted hallmark in this disease. In this review we summarize the latest findings in the mechanisms leading to the bone pathology in Gaucher disease in relationship with the proinflammatory state.

## 1. Osteoimmunology

A diverse number of interactions between bone and immune cells occur within the bone microenvironment. Bone and immune cells share the same progenitors residing in the bone marrow and these progenitors are under the effect of the same molecules including cytokines; these molecules can have a high influence in the hematopoiesis process, local immune responses, and bone cell development.

There is evidence that several immune cells can influence bone cell development and activity. However, the key players in this regulation are activated T-cells. After successful antigen-specific activation, T-cells produce a number of proinflammatory cytokines [1] that can act directly or indirectly on cells involved in bone turnover shifting bone balance towards bone resorption or bone generation.

The bone turnover process involves bone removal by resorbing osteoclasts and bone formation by osteoblasts. These processes are strictly regulated in physiological conditions, and this regulation implies the participation of osteocytes, which are the final step of osteoblast differentiation [2].

Osteoclasts are bone resorbing cells that derive from the same progenitors as macrophages and dendritic cells (monocyte/macrophage lineage) [3]. RANKL and macrophage colony stimulating factor (M-CSF) are essential for commitment of the common precursor to the osteoclast lineage and survival of differentiated osteoclasts. In addition numerous cytokines are also able to influence osteoclast differentiation and/or function [4].

Osteoblasts are the bone forming cells that originate from bone marrow-residing multipotent mesenchymal stem cells. Osteoblasts are one of the major sources of RANKL and in this manner they control bone resorption. These cells can influence immune cells and are critical regulators of the hematopoietic stem cells (HSC) from where immune and other blood cells derive [5].

RANKL is a transmembrane protein of the TNF superfamily encoded by the* Tnfsf11 *gene. It is expressed on the surface of osteoblasts (at different stages of differentiation), osteocytes, stromal cells of undefined origin, B- and T-cells, synovial fibroblasts, hypertrophic chondrocytes, and even osteoclasts themselves. The receptor of RANKL is RANK, which is encoded by the* Tnfrsf11a* gene. Upon stimulation of RANK by RANKL under costimulatory signals such as M-CSF, the process of osteoclast differentiation and maturation begins [6]. The third protein member of the osteoclastogenesis axis is called osteoprotegerin (OPG) and is encoded by the* Tnfrsf11b *gene. OPG functions as a soluble decoy receptor for RANKL, inhibiting RANKL interaction with RANK, thus acting like an antiosteoclastogenic molecule [7]. OPG is expressed by osteoblasts and other mesenchymal cells [8].

The RANK/RANKL/OPG axis is essential in osteoclast differentiation* in vivo* as mutations in genes encoding RANKL, RANK, or OPG lead to disorders with high bone pathology [9]. RANKL is presented in two different forms as a membrane-anchored molecule or as a soluble protein released by the action of matrix metalloproteinases [10]. Both forms of the protein have osteoclastogenesis activity; however, the membrane-anchored form functions more efficiently [11].

The expression of RANKL on mesenchymal cells, such as osteoblasts, is upregulated by osteoclastogenic factors such as vitamin D3, prostaglandin E2, parathyroid hormone, and several cytokines including IL-1, IL-6, IL-11, IL-17, and TNF-*α* [12].

## 2. Osteoimmunology in Pathological Conditions

The activation of immune cells is a requisite for defense of the host against pathogens; however, a persistent overactivation of effector cells under certain pathological conditions can result in tissue damage.

In the early 1980s, osteoclasts were identified throughout the synovium and at the synovium/bone interface in joints of rheumatoid arthritis (RA) patients [13]. These observations led to the determination that osteoclasts play an important role in certain pathological conditions [14, 15].

Inflammatory cytokines such as IL-1, IL-6, and TNF-*α* are present at high levels in the synovial fluid and synovium of RA patients. These cytokines have a potent capacity to induce the expression of RANKL on synovial fibroblasts and bone derived stromal cells and to affect osteoclast differentiation, thus directly contributing to the bone destruction process [13].

Osteoporosis (OP) has been traditionally considered as an endocrine disease resulting mainly from the estrogens decline after menopause. This change affects bone remodeling, leading to higher risk of fractures. Since the endocrine point of view by itself does not completely explain the pathogenesis of OP, the osteoimmunological approach raised and suggested that the production of proinflammatory cytokines such as TNF-*α*, IL-1, IL-6, IL-7, and IFN-*γ* by activated T lymphocytes could contribute to menopausal changes in bone dynamics [16, 17].

Phenylketonuria (PKU) is an inborn error of amino acid metabolism resulting from deficiency of phenylalanine hydroxylase, the key enzyme for phenylalanine metabolism. Bone impairment has been widely documented in PKU, using both radiological and ultrasound methods [18, 19], and it is typically associated with increasing age. In 2010 it was shown that PKU patients present increased numbers of circulating osteoclast precursors with higher differentiation potential compared to healthy controls. TNF-*α* levels and the RANKL/OPG ratio were increased in supernatants of PBMC cultures from patients and it was shown that the increased osteoclast differentiation from PBMC was RANKL dependent [20].

## 3. Gaucher Disease

Gaucher disease is the most prevalent lysosomal disorder [21], of around 1 : 13,000–60,000, and with a higher frequency in the Ashkenazi Jewish population [22]. Gaucher disease (GD; MIM #230800) is an autosomal recessive inherited lysosomal storage disorder that is due to a deficiency of glucocerebrosidase (acid beta glucosidase; GCase; EC 3.2.1.45). GCase deficiency results in progressive, intralysosomal accumulation of glucosylceramide in different tissues, primarily in cells of mononuclear-macrophage lineage. Lipid accumulation in macrophages results in engorged cells called “Gaucher cells.” Rarely, a variant GD may be secondarily caused by deficiency of the saposin C, the activator of the enzyme [23].

The first specific treatment for a lysosomal disorder was introduced for Gaucher disease, the enzyme replacement therapy (ERT) [24].

Clinical phenotype of GD reflects a continuum ranging from neuronopathic forms (GD types II and III) to the more frequent visceral form (GD type I) and from early onset to late onset [25].

Type I is observed in 90% of cases and is characterized by the lack of CNS manifestations. Clinical alterations are visceral (hepatosplenomegaly without organ disfunction), hematological (anemia and thrombopenia), and skeletal [26].

Bone pathology remains the main problem for GD I patients after the introduction of enzyme replacement therapy. Bone disease is a common and often painful and disabling manifestation of GD. Multiple compartments of bone that are affected are caused by alterations in bone metabolism (turnover, remodeling, and mineralization).

Almost all GD patients develop skeletal complications, consisting mainly of remodeling failure, osteopenia, osteoporosis, marrow infiltration, avascular necrosis, and osteolysis [27]. It may be suggested that patients with early onset GD I are at risk of skeletal disease. One of the early signs is the typical “Erlenmeyer flask” deformity of the distal femur. These changes predominantly affect long bones and the vertebrae. Patients could be asymptomatic with or without radiological signs or present symptoms including bone pain involving one limb or joint, avascular necrosis, or pathological fractures. An international registry of Gaucher patients worldwide revealed that 62% of them had some form of radiologic bone disease and 43% experienced bone pain [28]. The M*ϕ* are prominent in the bone marrow and contribute to acute episodes of osteonecrosis, particularly during growth. Necrosis of the marrow leads to impaired function of joints. Other effects on the skeleton include local swellings known as Gaucheromas.

Imaging methodologies for the evaluation of skeletal involvement, such as conventional (plain) radiography and scintigraphy, MRI, computed tomography, or dual energy X-ray absorptiometry, are currently employed and provide accurate evaluation and staging of bone lesions in GD [29].

Much evidence demonstrates substantial improvement of hematological and visceral parameters upon introduction of specific ERT for Gaucher patients [30]. However, bone tissue does not respond equally; it is, in some degree, refractory to therapy. Patients at risk may benefit from early intervention with ERT, although many lesions and osteonecrosis are irreversible. Enzyme therapy cannot reverse established osseous injury [31]. Several prospective studies have been performed to evaluate the effectiveness of ERT in treating skeletal pathology. Bone pain is present at baseline in around two-thirds of the patients. Some patients improve in this aspect, but 40% of patients remain with this symptom after 18 months of treatment. In a recent study of patients treated with imiglucerase for 10 years, a positive effect was observed in skeletal symptoms, as well as a reduction of bone pain and crises in patients who suffer from them at baseline. Moreover, most of the patients who did not report bone symptoms at baseline continued to be pain-free after 10 years of ERT [32].

Bone mineral density tends to increase during therapy, but the response is slow [31]. Patients with preexistent skeletal complications tend to suffer incidents during ERT, such as medullary infarctions, avascular necrosis, or fractures, but the frequency of these events is reduced [33]. Low bone density manifests early in children with GD, and mineral density deficit is maximal in the adolescent period. Moreover, this group is most responsive to ERT, underscoring the importance of early diagnosis and intervention to achieve optimal peak bone mass [34]. In the largest study with treated pediatric patients bone mineral health was impaired in a large proportion of the group before ERT and improved considerably with treatment [35].

## 4. Inflammation in Gaucher Disease

A chronic stimulation of the immune system is a well-accepted hallmark in GD. Studies of the proinflammatory state in patients were mainly focused on analyzing cytokine levels in sera [36–38]. Although there is a high variation among patients, increased levels of IL-1*α*, IL-1*β*, IL-1Ra, sIL-2R, IL-6, IL-8, IL-10, IL-18, TNF-*α*, TGF-*β*, M-CSF, MIP-1, and CCL18 have been reported in sera [39–41].

Macrophages (M*ϕ*) are the principal cell type compromised in patients with GD. M*ϕ* have several different functions including tissue remodeling and host defense; on the other hand they play central roles in many disease processes. They can secrete both anti- or proinflammatory cytokines depending on the activation signals. Upon activation, two main phenotypes of M*ϕ* could be produced: classical or alternative, depending on environment present at the time of the stages of activation [42]. Gaucher cells resemble alternative activated M*ϕ* [43], characterized by the expression of chitotriosidase and CCL18.

Several immune cells have been shown to be impaired in GD including monocytes, M*ϕ*, dendritic cells, and T- and B-cells [44–46]. It has been shown that monocytes in GD patients expressed higher levels of CD1d and MHCII on their surface, which could lead to an increased T-cell activation [47]. Abnormalities in the B-cell subset are mainly IgG and IgM hypergammaglobulinemia and plasmacytosis [48]. An increased incidence of gammopathies and multiple myeloma has been reported, further showing the interplay between Gaucher cells and the immune system [49].

A GD murine model was generated, in which the* GBA *gene was conditionally deleted on hematopoietic cells [50]. This model presented all the hallmark characteristics of GD I, including organomegaly, and it was the only murine model so far to show bone involvement. In this model an alteration of immune cell compartment was observed. This alteration included thymic maturation impairment with higher levels of CD4^+^ and antigen-presenting cells. What is more, activated B-cells on the thymus were also increased, which could explain the alteration of normal T-cell maturation [51].

In another murine model of GD, higher levels of CD4^+^ cells were found on the lungs, spleen, and liver as well as an increased expression of costimulatory molecules [52]. Higher levels of proinflammatory cytokines including IFN-*γ*, IL-12p40, TNF-*α*, IL-17A/F, IL-6, and TGF-*β* were found in sera of these mice. When T-cells were cocultured with dendritic cells in the presence of glucosylceramide, higher levels of Th1/Th17 cytokines were secreted.

More recently, using a different approach, Panicker et al. differentiated M*ϕ* from patient induced pluripotent stem cells (hiPSC); with this model they showed increased production of IL-1*β*, TNF-*α*, and IL-6 by GD derived M*ϕ* and an exacerbated response to LPS treatment [53].

This deregulation of immune system cells is tightly related to the increased levels of cytokines and chemokines. These molecules are secreted by the immune cells, which, in turn, are recruited and activated by chemokines and cytokines, respectively. This could create a loop in which immune cells from Gaucher patients are being continuously activated, leading to systemic and focal activation of the immune system.

## 5. Osteoclast-Osteoblast Uncoupling in Gaucher Disease

The molecular and cellular bases of GD bone physiopathology are not well understood and opposing studies have emerged in the last few years. As mentioned before in 2010, Mistry et al. [50] generated a conditional KO mouse model of GD I which presented the main GD clinical hallmarks. The most striking feature about this model is the presence of bone involvement as previous mouse models of GD did not present bone involvement. Bone manifestations included medullar infarctions with associated avascular necrosis and osteopenia at all sites. The bone formation rate presented a significant impairment in these mice while the quantification of TRAP-labeled surfaces did not present differences.

A significant impairment in osteoblast proliferation and differentiation was present in the model, while osteoclast differentiation and activity did not seem to be altered. The impairment on osteoblast proliferation was shown to be dependent on a decrease in PKC activity due to the accumulation of glucosylsphingosine and, to a lesser extent, glucosylceramide. More recent studies present sphingosine as the most probable candidate for osteoblast impairment in the mouse model [54]. These findings suggest that bone complications in GD would result from an osteoblast source without osteoclast involvement [50, 54].

Different reports have shown the involvement of osteoclasts on GD bone pathophysiology. Using an* in vitro* model of GD in which mesenchymal stem cells and monocytes were exposed to conduritol-*β*-epoxide (CBE), a specific glucocerebrosidase inhibitor, Lecourt et al. showed that although direct CBE treatment had no effect on osteoclast differentiation if mesenchymal stem cells were cultured in the presence of conditioned media from CBE-exposed monocytes, an increased osteoclastogenesis and resorption activity was detected [55].

Our group showed, using a similar approach, that treatment of osteoclast precursors with conditioned media from peripheral blood mononuclear cells (PBMCs) exposed to CBE resulted in an increased level of osteoclast differentiation when compared to control conditioned media.

What is more, we showed that one of the central molecules involved in the increased osteoclast differentiation was the proinflammatory cytokine TNF-*α* and that T-cells also played an important role in this process [56]. The same results were obtained using a mice model in which conditioned media were obtained from peritoneal M*ϕ* or splenocytes exposed to CBE; in this model involvement of TNF-*α* was also shown using osteoclast precursors derived from TNF-*α* receptor deficient mice [57]. In addition to this we could show that treatment of the osteoblastic cell line MC3T3 with conditioned media from CBE treated M*ϕ* reduced mineralization and collagen deposit [57]. These results would indicate an impairment of both osteoclast and osteoblast activity in GD leading to bone loss as the involvement of immune cells and molecules in this process.

The group of Reed et al. isolated PBMC from patients with GD and showed that patients' monocytes, when exposed to osteoclastogenic mediators, presented a higher differentiation towards active osteoclasts. What is more, osteoclasts differentiated from patients had bigger diameter and a greater number of nuclei when compared with osteoclasts differentiated from healthy controls' PBMCs. They showed that the higher osteoclastogenic potential presented a clinical correlation with patient's bone involvement [58].

## 6. Future Perspectives

GD is the most common lysosomal disorder and the first for which specific treatment has been developed. Bone disease in Gaucher patients is one of the most disabling features of the disease, so the possibility of knowing the mechanisms underlying the bone pathology is a main challenge to ameliorate the quality of life of patients.

Studies are based on the explanation of the cellular and molecular pathways that result upon glucosylceramide accumulation in M*ϕ* and the possible relationship with different bone cells.

The bases of osteoimmunology are being applied to bring light in this aspect. In this regard, there are several questions to be answered. T-cell involvement and a better understanding of the effects and importance of proinflammatory cytokines such as TNF-*α* on bone pathology in GD are necessary.

On the other hand, crosstalk between osteoblasts and osteoclasts in GD could provide new mechanisms involved in the process of bone loss.

Finally the effect of ERT and substrate reduction therapy on bone involvement is a central aspect to be studied, especially, how these treatments affect different bone cells and their function.

The results of basic research will be of utility in order to identify new targets for coadjuvant therapies to treat skeletal pathology in GD.
